# Non-operative Management of an Accidental Knee Intra-articular Nail

**DOI:** 10.7759/cureus.37499

**Published:** 2023-04-12

**Authors:** Jennifer Kordell, Jennifer Cogburn, Morteza Khodaee

**Affiliations:** 1 Family Medicine, University of Colorado, Denver, USA

**Keywords:** nail barb, penetrating objects, occupational injuries, arthrotomy, nail gun

## Abstract

Nail gun injuries are relatively common presentations to the emergency department. The majority of these injuries occur to the hands and rarely result in long-term morbidity. However, despite the large number of cases each year, little research is available regarding the optimum emergency management of nails that implant intra-articularly. Initial studies suggested that cases of nails penetrating intra-articular or neurovascular structures warranted operative debridement; however, newer studies have suggested cautious nail removal, wound debridement, irrigation, antibiotic coverage, and tetanus prophylaxis are equivalent to operative intervention for the management of most intra-articular nails. We present a gentleman in his 40s with accidental penetration of a nail fired from a nail gun into his right knee. He was neurovascularly intact. After initial evaluation and management, he was transported to a higher level of care for operative management. However, the nail was ultimately removed bedside utilizing adequate anesthesia.

## Introduction

Nail guns, a mainstay in the commercial and residential construction industries, have the potential to cause considerable injury or even death, firing nails at up to 400 meters/second [1]. It is estimated by the Centers for Disease Control and Prevention (CDC) that nail guns are responsible for approximately 37,000 emergency department (ED) visits each year [2]. However, despite the large number of cases, little research is available regarding the optimum emergency management of nails that implant intra-articularly [1,3-6]. We present and discuss the evaluation and management of a patient with an accidental knee intra-articular nail gun injury.

## Case presentation

**Figure 1:**
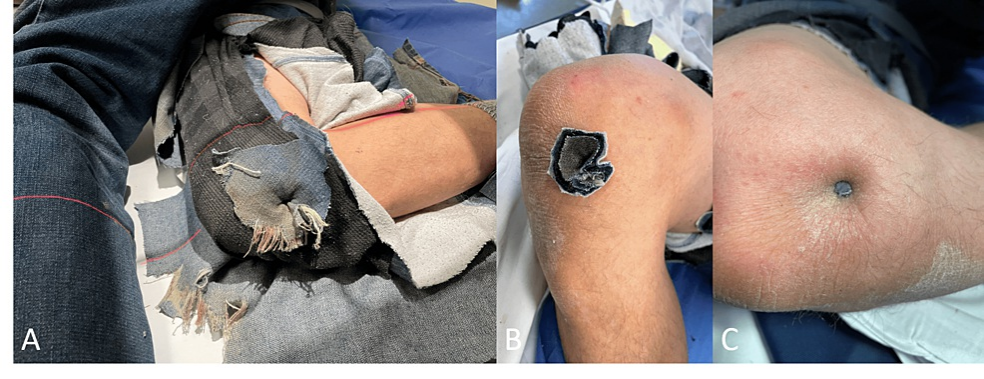
Patient presented with the right knee joint fixed in a 90° flexed position (A). The long nail was embedded into the anteromedial aspect of the right knee and after the removal of the cloth, the wound appeared fairly clean (B, C).

A gentleman in his 40s presented to an ED (a remote facility without access to a surgical operative room) after accidentally firing a long nail from his nail gun into his right knee. On presentation, although neurovascularly intact, the patient was non-weight-bearing on the limb, and the knee joint was fixed and flexed to 90° (Figure 1A). The long nail embedded itself into the anteromedial aspect of the right knee. The wound appeared fairly clean (Figures 1B, 1C).

**Figure 2:**
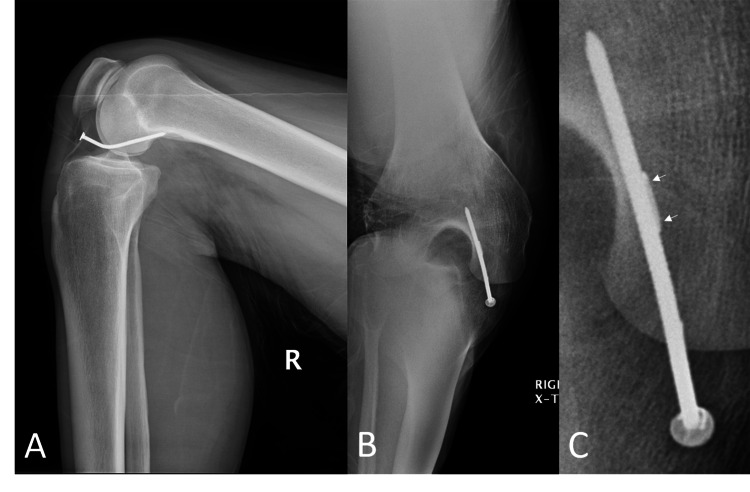
Plain radiography revealed a penetrating nail entering the medial aspect of the knee, just medial to the patellar tendon, and entering the medial femoral condyle (A, B). In magnified view, the nail barb (arrows) is visible (C).

X-ray imaging showed the nail entered the medial intra-articular space missing the tibial plateau and patellar tendon and bent cephalad into the medial femoral condyle (Figure 2).

On initial presentation, due to the lack of access to the required medical instrument and operative room in the vicinity, the nail was determined to be manually irremovable without operative management. Therefore, the patient was given tetanus prophylaxis, intravenous cefazolin, ketamine for sedation, and fentanyl for pain control. He was then transported to a higher level of care. He ultimately underwent bedside nail removal utilizing intra-articular, intravenous, and local anesthesia, and his wounds were copiously irrigated.

**Figure 3:**
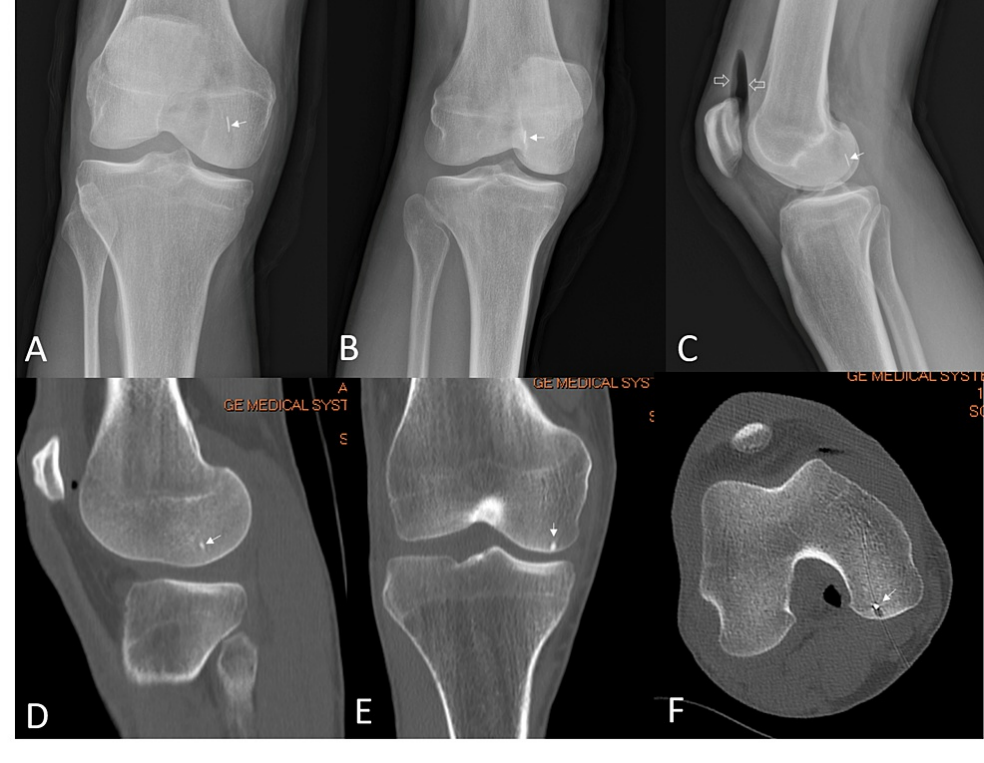
Post-nail removal X-rays (A-C) and CT scan images (D-F). Lateral view (C) showing intra-articular air (open arrows) as a result of irrigation.

Post-nail removal X-rays (Figures 3A-3C) and CT scan images (Figures 3D-3F) showed a small 7 mm retained foreign body, likely a barb from the nail, embedded in the medial femoral condyle deep to the subchondral bone plate.

Due to the intraosseous and not intra-articular nature of the barb, it was determined that the patient would not benefit from attempted operative foreign body removal. The patient was discharged on oral cephalexin for seven days with strict return precautions. The patient never experienced any complications on interval examinations.

## Discussion

Despite adding many safety features, nail gun injuries are still relatively common presentations to the ED. A review by the CDC showed these injuries most commonly involve the non-dominant hand/fingers (58%) and lower extremities (24%) [2]. A review by Horne et al. of 88 nail gun injuries showed no significant vascular or neurological injuries that required repair, and only a 3.4% infection rate [3]. Notably, infections were limited to those who presented more than one day after injury [3]. Given the prevalence of nail injuries, under proper circumstances, front-line trauma providers should feel empowered to remove nails under local anesthesia without referring the patients to the operating room.

On initial presentation, a detailed physical examination is of utmost importance. The site of penetration should be closely evaluated for evidence of open fractures, gross deformity, limitations in range of motion, and proximity to neurovascular structures [1,6]. Radiographs with a minimum of two views of the injured site should always be obtained on presentation and after nail removal [1,5,6]. These radiographs must be scrutinized for associated fractures, joint penetration, and the presence of metallic barbs or other possible retained foreign bodies [1,5,6]. Capillary refill and two-point discrimination are needed to assess neurovascular status pre- and post-nail removal. Grossly contaminated wounds, absent pulses, loss of sensation, persistent intra-articular involvement, unstable fractures, and tendon injuries should be closely evaluated, as they may warrant surgical exploration [1,5,6]. Otherwise, local debridement, tetanus vaccination, and early intravenous antibiotics followed by a subsequent course of oral antibiotics are shown to be safe and effective in the management of nail gun injuries [1,5-7].

## Conclusions

Patients presenting with an uncomplicated intra-articular nail without neurovascular compromise should undergo a bedside attempt for removal in contrast to the commonly used operative methods. This may be more applicable if access to a higher level of care is limited and difficult. Non-operative methods are shown to be adequate to remove nails that are not paired with contaminated wounds, absent pulses, loss of sensation, unstable fractures, tendon injuries, and retained intra-articular foreign bodies. Providers should, at least, have all the necessary instruments to attempt the removal. In most cases, removal requires a significant amount of force and adequate anesthesia is essential. Tetanus prophylaxis, cautious nail removal, local debridement, irrigation, and prophylactic administration of a first-generation cephalosporin for seven days should be appropriate management in most cases.
